# Network and stoichiometry analysis revealed a fast magnesium and calcium deficiency of mulched *Phyllostachys violascens*

**DOI:** 10.3389/fpls.2024.1492137

**Published:** 2024-11-27

**Authors:** Hanchang Zhou, Siyuan Huang, Ziying Zhang, Ting Li, Yi Li, Guoqiang Zhuang, Guohua Liu, Bojie Fu, Xiaobao Kuang

**Affiliations:** ^1^ The Bamboo Institute, Jiangxi Academy of Forestry, Nanchang, China; ^2^ The Research Centre for Eco-environmental Sciences, Chinese Academy of Sciences, Beijing, China

**Keywords:** organic materials mulching, Lei-bamboo, rhizosphere microbial community, stoichiometry, magnesium, calcium

## Abstract

The imbalanced fertilization and the consequential deterioration on the rhizosphere microbial community (RMC) were two potential reasons for the quick yielding degradation of *Phyllostachys violascens* (Lei-bamboo), a high-value shoot-oriented bamboo. However, most research only focused on nitrogen, phosphorus, and potassium; the studies on the dynamics of other nutrients, such as calcium and magnesium; and their driving mechanisms, lags far behind. Thus, Lei-bamboo fields of different mulching and recovery ages were selected to investigate the dynamics of calcium and magnesium in both soil and bamboo tissue, and to explore their relationship to RMC composition and network patterns. The results showed that mulching increased the content of soil acidification, total organic carbon, alkali-hydrolysable nitrogen, available phosphorus, and available potassium but reduced soil exchangeable magnesium and calcium in soil as well as the magnesium and calcium content in rhizome, stem, and leaf of Lei-bamboo, which indicated an increased relative limitation on magnesium and calcium. Mulching also enhanced the α-diversity and reshaped the composition of RMC, which had a close link to Mg rather than nitrogen, phosphorus, and potassium. As the mulching years increased, the RMC network became bigger and more complex, and the magnesium and calcium gradually appeared in the network center, which further support the magnesium and calcium deficiency to RMC. Nearly all the variation mentioned above could be revered after the removing of mulching. Structural equation modeling showed two main pathways that mulching leads to magnesium and calcium deficiency in Lei-bamboo, one is directly by lowering soil magnesium and calcium content, the other one is indirectly by improving RMC network interactions, a sign of weakened mutualism between RMC and plant roots that hampering the uptake of nutrients. This research highlights the quick magnesium and calcium deficiency caused by mulching in Lei-bamboo forest and the contribution of RMC in amplify the effects of soil magnesium and calcium deficiency, which offers valuable information on balancing fertilization pattern for future sustainable Lei-bamboo cultivation.

## Graphical Abstract

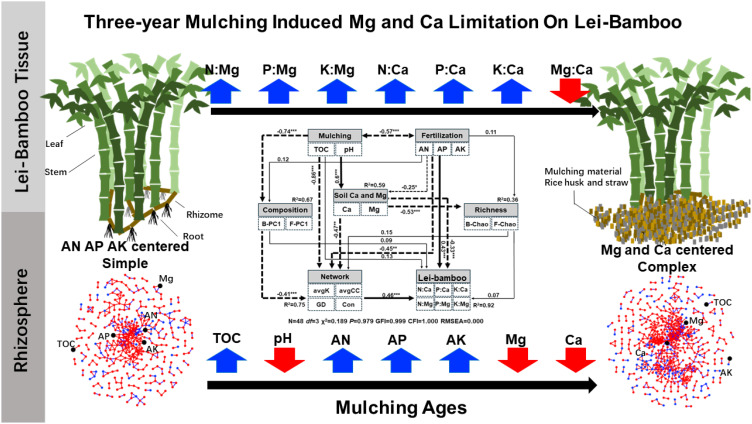

## Highlights

- Mulching induced magnesium and calcium deficiency in soil and plant tissue.
- Mulching enhanced rhizosphere microbial network interactivity.
- Microbial network focus more on magnesium and calcium as the mulching age increases.
- Increased network interactivity might amplify the effects of soil magnesium and calcium limitation.

## 1 Introduction

*Phyllostachys violascens* (Lei-bamboo) is a high-value and food-oriented bamboo species that exhibits a high potential for enhancing the local people’s incomes (Gui et al., 2013; Chen et al., 2015). Most farmers adopt intensive cultivation pattern to ensure the yielding and income from Lei-bamboo forest (Wei et al., 1994; Gui et al., 2018). The intensive cultivation of Lei-bamboo includes not only classic intensive measures, such as high-quantity fertilization with nitrogen, phosphorus, and potassium, but also with organic material-based mulching, a special intensive measure of Lei-bamboo cultivation (Wei et al., 1994; Gui et al., 2013; Chen et al., 2015). For about 4 months, beginning in November, the soil is covered with a mixed layer of straw and rice husks, 20–30 cm thick, to increase the soil temperature and stimulate the sprouting of shoots, thereby increasing shoot yield (Wei et al., 1994). Mulching can potentially increase yield by as much as 3 times and raise the yield to as high as 375,00 kg ha^−1^ (Gui et al., 2013), the harvest income can reach nearly USD 30,000 ha^−1^ in certain regions (Chen et al., 2015). However, after several years of mulching, the yield will sharply decrease, especially after the third year (Gui et al., 2013), and even if fertilization is doubled, the yield could still be decreased by as much as 50% (Wang et al., 2017). Only after 2 years of recovery cultivation, with no mulching, can Lei-bamboo again achieve a considerable yield using the mulching technique (Wang et al., 2017). Moreover, after long-term intensive cultivation (usually over 15 years), Lei-bamboo is incapable of yield recovery through mulch removal. Full recovery can only be achieved by exchange of the soil to a depth of more than 1 m and replanting of the bamboo (Zhou et al., 2017). The undesirable effects caused by mulching not only reduce the potential yield but also increase management costs, in particular, the rapid yield reduction after just 3 years of mulching has become a core problem to be resolved in Lei-bamboo intensive cultivation (Wang et al., 2017; Zhou et al., 2017). Unfortunately, the detailed degradation mechanisms for mulched Lei-bamboo forests were still unclarified.

Empirically, mulching-induced impairment of soil nutrient cycling and subsequent deterioration of the rhizosphere microbial environment have been hypothesized to be factors for the short-term degradation of Lei-bamboo (Jiang et al., 2000; Dai et al., 2018; Das et al., 2021). However, most studies have only focused on the nutrient cycling and microbial processes of nitrogen, phosphorus, and potassium applied under mulching and on their relationship to yield degradation (Wei et al., 1994; Gui et al., 2013; Chen et al., 2015), with the variation of the content and stoichiometric of other elements in soil and Lei-bamboo tissue, such as magnesium and calcium, scarcely discussed. It seems that it had been tacitly assumed that magnesium and calcium were relatively adequate and less affected by mulching. Magnesium and calcium play important roles in photosynthetic (Jing et al., 2024) and organic matter transportation and storage for plants (Farhat et al., 2016). Magnesium and calcium can also influence the soil biogeochemical processes and the effects of nitrogen and phosphorus fertilizer and their. The application of magnesium and calcium were reported to save half nitrogen fertilizer in a consecutively cultivated peanut field (Gao et al., 2024b) and can reduce the N_2_O emission and nitrate leaching so that to enhance nitrogen fertilizer efficiency in a paddy soil (He et al., 2023). Hence, the magnesium and calcium limitation could both directly and indirectly contribute to the short-term degradation of Lei-bamboo and lower the yielding.

Yet, the dynamic of magnesium and calcium through mulching years and whether they were limiting factors of mulched Lei-bamboo forests are still open questions, as mulching may increase or decrease the availability of soil magnesium and calcium (Clark et al., 2005; Yang et al., 2010; Prietzel et al., 2021). The availability of soil magnesium and calcium was determined by mineralization and leaching, two processes mainly regulated by microbial activity and soil acidity. Mulching could offer controversial influences on these two processes. Mulching vegetable plots with straw, for example, releases organic matter that adsorbs free protons from the soil and subsequently mitigates soil acidification (Guan et al., 2022; Le et al., 2022), while some studies have found that mulching materials can release organic acids and accelerate soil acidification (Gui et al., 2018). Mulching may introduce anaerobic stress (Qian et al., 2022) and aluminum toxicity stress (Beeckman et al., 2018; Zhao et al., 2020), reducing the activity of microbial communities and their ability to turn over nutrients, but mulching also releases organic matters that promote microbial community activity and subsequently accelerate mineralization (Guan et al., 2022; Le et al., 2022). Thus, it’s necessary to systematically investigate the dynamic of magnesium and calcium along a mulching cycle of Lei-bamboo and introduce stoichiometry to test their relative limitation to shoot yields. Stoichiometry ratio is a commonly used tool to evaluate the relative limitation of certain nutrients, the increase and decrease of the ratio indicate an uplifting limitation on denominator nutrient and numerator nutrient, respectively (Martiny et al., 2013).

The root is the core plant organ for magnesium and calcium assimilation, and the rhizosphere soils are thus more sensitive to nutrient limitation variation. Meanwhile, rhizosphere is the location of a highly active microbial community, named the rhizosphere microbial community (RMC) (Ling et al., 2022). The RMC receives organic exudates from their host plants and, in return, aides the host with nutrient assimilation (Pausch et al., 2024), pathogen defense (Trivedi et al., 2020), and stress tolerance (Aslam et al., 2022). As the bridge linking host plant and bulk soil, the properties of the RMC, including richness, composition (Vorholt et al., 2017), and network interactions (Jiang et al., 2017), are regulated by both the physiological signals of host plants (e.g., certain nutrient deficiencies) (Venturi and Keel, 2016) and the soil physiochemical properties (e.g., nutrient contents and their limitations) (Garbeva et al., 2008). The RMC is sensitive to environmental change and can quickly reshape richness, composition, and network interactions accordingly, and it can drive host plant community variation (Zhou et al., 2021, 2022). The RMC community structure is largely regulated by the most critical limiting factors, and the network interactions will also tend to be structured around these factors (Zhou et al., 2021, 2022). Thus, changes in Lei-bamboo RMC may indicate variations in both soil physiochemical properties and plant nutrient physiological status of magnesium and calcium, which possibly offers a deeper explanation of the interactive mechanisms between the latter two (Jiang et al., 2017; Vorholt et al., 2017).

In this study, the rhizosphere soil physiochemical properties, plant nutrient content, and RMC properties of Lei-bamboo with different mulching and recovery ages were investigated to explore the dynamics of magnesium and calcium in soil and plant tissue caused by mulching and to resolve the relevant mechanisms. Given the fact that the 20–30 cm thick organic mulching layer would deliver large amounts of organic matter to the soil, we hypothesized that mulching would increase soil pH and increase soil available magnesium and calcium content. However, because a large amount of nitrogen, phosphorus, and potassium was applied before mulching, we hypothesized that the relative limitation of magnesium and calcium in both soil and Lei-bamboo tissue would increase, indicated by an increase in the corresponding stoichiometry (e.g., N:Mg), which would lead the RMC networks under mulching focused more on magnesium and calcium rather than on N, P, and K. The mulching should offer a more hospitable environment for microorganisms, as it introduces a considerable quantity of foreign organic matters, which would not only enhance interactions among microbes but also weaken the functional link between root and RMC. Thus, we hypothesized that mulching would increase both RMC diversity and network interactivity. Last, the increased relative limitation on soil calcium and magnesium and the weakened cooperation between root and RMC combined to induce calcium and magnesium deficiency in Lei-bamboo under mulching conditions. This research further highlighted the importance of magnesium and calcium in Lei-bamboo cultivation and offered new sight to protect mulched Lei-bamboo forest from short-term degradation.

## 2 Materials and methods

### 2.1 Location and sampling

The target Lei-bamboo fields were in Hongxi Farm of Fuzhou city, Jiangxi province, China (E 116°20′37.17″, N 28°27′03.46″). The site is a mid-subtropical zone, with a mean annual temperature of 19.3°C and mean annual precipitation of 1,742 mm. The bamboo was planted from 2014 to 2019, and samples were taken on 5 March 2023 (the mulching materials had recently been removed), when the farm possessed a bamboo field that would be mulched in the next year (M0, planted in the winter of 2019), a field that had been mulched once (M1, planted in the winter of 2018), a field that had been mulched for two consecutive years (M2, planted in the winter of 2017), a field that had been mulched for three consecutive years (M3, planted in the winter of 2016), a field that had recovered for one year (R1, planted in the winter of 2014), and a field that had recovered for 2 years (R2, planted in the winter of 2015). Every field was large enough (over 6,660 m^2^) to ensure representativity. All fields were selected from the same farm to reduce variations introduced by climate conditions and soil parent materials. Mulching operations are initiated in early November when a mixture of rice husks (200t hm^−2^) and straw pieces (50t hm^−2^), with a thickness of over 25 cm, is placed over the bamboo growing beds. The mixture contains about 0.06% nitrogen, 0.015% phosphorus, 0.12% potassium, 0.24% calcium, and 0.14% magnesium. This mulch is removed approximately 4 months later. About 7.5 kg year^−1^ m^−2^ organic fertilizer was applied to the fields in the middle of November, and the middle of March, respectively. About 0.188 kg year^−1^ m^−2^ chemical fertilizer (N:P:K = 10:3:7) was applied to the fields in early March, early June, early September and mid-November. The soil belongs to subtropic soils (Latosol), which developed from river sediments, the bulk density of the field soil is about 1.29 g cm^−3^.

During sampling, eight 5 m × 5 m plots were set in each field, the fine roots of the rhizomes within the square were dug up and bulk soil removed by shaking, then the adhering rhizosphere soil (mainly 30 cm deep topsoil of where roots were distributed) was collected by brushing. After all the rhizosphere soil in the square was collected, 100 g were retained for analysis. The rhizomes, stems, and leaves in the square were respectively collected and crushed, and approximately 1 kg was retained for analysis. The samples were transported to the lab on ice. 5 g of each soil sample were stored at −80°C for molecular analysis. The remaining soil was air dried, grounded to pass through a 0.2-mm sieve, and stored at −80°C until measurement of physiochemical properties. The plant tissue samples were dried to a constant weight at 100°C, then the tissue was powdered to pass a 0.2-mm sieve, and approximately 100 g were stored in a drying dish at room temperature.

### 2.2 Soil physiochemical properties

The total organic carbon (TOC) was measured by a TOC analyzer (Elementar, Berlin, Germany). 0.1g sample was weighed and then burned at 950°C with gas flow 250 ml min^−1^ (Contreras et al., 2018).

The available phosphorus (AP) was determined by the Mo-Sb-Vc method. 2.5 g soil sample was placed in a 50-ml centrifuge tube, to which 18 ml extraction solution (0.03 mol L^−1^ NH_4_F and 0.025 mol L^−1^ HCl) was added. The mixture was then shaken at 300 rpm for 1.5 min. 5–10 ml of the supernatant was transferred to another bottle, to which 5 ml Mo-Sb-Vc solution (100 ml 5 g L^−1^ C_8_H_4_K_2_OSb_2_, 450 ml 22.22 g L^−1^ (NH_4_)_2_MoO_4_, 10.5 g Vitamin C, and 153 ml concentrated sulfuric acid) was added and deionized water to adjust the total volume to 50 ml. The solution was incubated at 25°C for 30 min, and the 880 nm luminous absorbance value was then measured by a spectrophotometer (Simatsu-UV1900, Tokyo, Japan). The AP content was subsequently calculated (Huang et al., 2020).

The available potassium (AK), exchangeable magnesium (Mg), and exchangeable calcium (Ca) were measured by an atomic spectrum spectrophotometer (Spectrum-3803AA, Shanghai, China). A 2.0 g soil sample was placed into a 50 ml centrifuge tube, and extraction solution (1 mol L^−1^ NH_4_Ac, pH = 7.0) was added. Next, the solution was shaken at 300 rpm for 30 min, and the supernatant was collected. AK, magnesium, and calcium were determined at 766.5 nm, 285.2 nm, and 422.7 nm, respectively. The burning gas was ethyne (purity > 99.99%) (Kurokawa et al., 2020).

Alkali-hydrolysable nitrogen (AN) was measured by diffusion method (Chen et al., 2016). The pH was determined by a pH meter (Leici-PHS3G, Shanghai, China), where 5 g of soil was mixed with 12.5 ml of water, and the mixture was used for measurement (Zhou et al., 2022).

### 2.3 Plant nutrient content

For nitrogen content measurement, 1 g tissue powder was placed in a digesting tube, to which 1 g accelerator (K_2_SO_4_:CuSO_4_:Se = 100:10:1, thoroughly powdered and mixed) and 5 ml concentrated sulfuric acid were added. Measurement was performed using a Kjeldahl Nitrogen Analyser (Hanon K9860, Jinan, China) (Chen et al., 2016).

For measurement of phosphorus, potassium, calcium, and magnesium, 0.2 g plant tissue powder were placed in a digestion tube, and 8 ml of concentrated sulfuric acid was added. The mixture was allowed to sit for 12 h, after which it was digested at 250°C for 10 min, then heated to 380°C and 2 ml 30% H_2_O_2_ was added, the solution was digested until it became clear. After cooling to room temperature, the solution was transferred to a centrifuge tube, and water was added until the total volume reached 50 ml. Phosphorus content was determined by Mo-Sb-Vc method, and the potassium, calcium, and magnesium were measured by an atomic spectrum spectrophotometer (same as Section 2.2).

### 2.4 Rhizosphere microbial community composition and network

Total soil DNA was extracted by FastDNA-SPIN Kit for Soil (MP Biomedicals, Santa Ana, CA) according to the manufacturer’s instructions. The PCR primer pairs used were 338F-(5’-ACTCCTACGGGAGGCAGCA-3’) and 806R-(5’-GGACTACHVGGGTWTCTAAT-3’, targeting the bacterial 16S V3-V4 region (Liu et al., 2018), and ITS1F-(5’-CTTGGTCATTTAGAGGAAGTAA-3’) and ITS2R-(5’-GCTGCGTTCTTCATCGATGC-3’), targeting the Fungal ITS2 region (Adams et al., 2013). The PCR system was 50 μl, and included 1–10 ng DNA template, 1 μl forward primers and 1 μl reverse primers, 25 μl 2×Premix Taq, and enough water to bring the volume to 50 μl. The PCR program was 95°C pre-denaturing for 3 min, 40 cycles of (95°C denaturing for 30 s, 58°C annealing for 30 s, 72°C elongating for 30 s), 72°C for 8 min, then held at 4°C.

### 2.5 Statistic and data availability

#### 2.5.1 The taxonomical classification and abundance

Sequencing was conducted using an Illumina Novaseq6000 (Guangdong Magigene Biotechnology Co., Ltd. Guangdong, China). Using a sliding window (-W4-M20), the quality control on raw sequencing data was done by FASTP v0.14.1. Then the primers were removed by the CUTADAPT software program. Paired-end clean reads were combined using the usearch-fast_mergepairs command according to the overlapped reads. Chimeric sequences were removed using the usearch-cluster_otus command. Sequences with at least 16 bp overlap reads and less than five mismatch sites were merged. The merged sequences with similarity > 97% were defined as belonging to the same OTU (Edgar, 2013). The bacterial and fungal OTUs were annotated using Silva-138 (Prüsse et al., 2011) and Unite-v8.0 (Abarenkov et al., 2010), respectively. The OTU tables were resampled to account for sequencing depth, with the bacterial OTU table containing 45,326 reads for each sample and the fungal table containing 19,344 reads, and the resampled OTU tables were used for subsequent analyses.

#### 2.5.2 The calculation of alpha and beta diversity

The calculation of relative abundance (RA), α- and β-diversity, and the redundancy analysis/ canonical correspondence analysis (RDA/CCA) and analysis of similarities (ANOSIM) analyses (Bray-Curtis distance based) were all conducted using an online platform (http://cloud.magigene.com/, accessed on 14 February 2024). Chao1 and Shannon index were chose to assess alpha diversity. The difference in the microbiome composition was calculated using redundancy analysis/classical canonical analysis based on Bray-Curtis distances.

#### 2.5.3 The network analysis

The network analysis was conducted using an online platform (http://ieg4.rccc.ou.edu/MENA, accessed on 16 February 2024); the network construction parameters were set as majority=6;missing_fill=fill_paired(0.01);logarithm=n;similarity=spearman2, the difference of cutoff values among networks was less than 0.01 (Deng et al., 2012). Four parameters were calculated to quantify the structural variations of the network: the average degree (avgK), which indicates the network interactivity; the geographical distance (GD), which indicates the length of the interaction chain; the average clustering coefficient (avgCC), which indicates the network clustered state; and the connectedness, which indicates the number degree of the nodes connected (Deng et al., 2012). The identity of key nodes, including module hubs, the nodes that were potential functional centers of a module (Zi > 2.5), and connectors, the nodes that were key for interaction among modules (Pi > 0.625), were determined (Deng et al., 2012). The figures were drawn by Origin 8.0, and the networks were visualized by Cytoscape v3.3.0.

#### 2.5.4 The structure equation modeling analysis

The correlation analysis and between group differences analyses (one-way analysis of variance, Tukey’s HSD) were performed using IBM SPSS Statistics 27. The structural equation modeling (SEM) was performed by Amos v2.0 compounded in SPSS. During the reduction of data dimension for SEM, the main axis was significantly related to the factor’s axis (each coefficient higher than 0.8). The composition of the SEM was adjusted until the goodness of fit index (GFI) was over 0.9, the comparative fit index (CFI) was over 0.9, the root-mean-square error of approximation (RMSEA) was less than 0.05, the χ^2^/*df* was less than 2, and the *P*-value was over 0.05, and then chose the models of the best parameters (Weston and Gore, 2006).

## 3 Results

### 3.1 The influence of mulching on soil physiochemical properties

**Figure 1:**
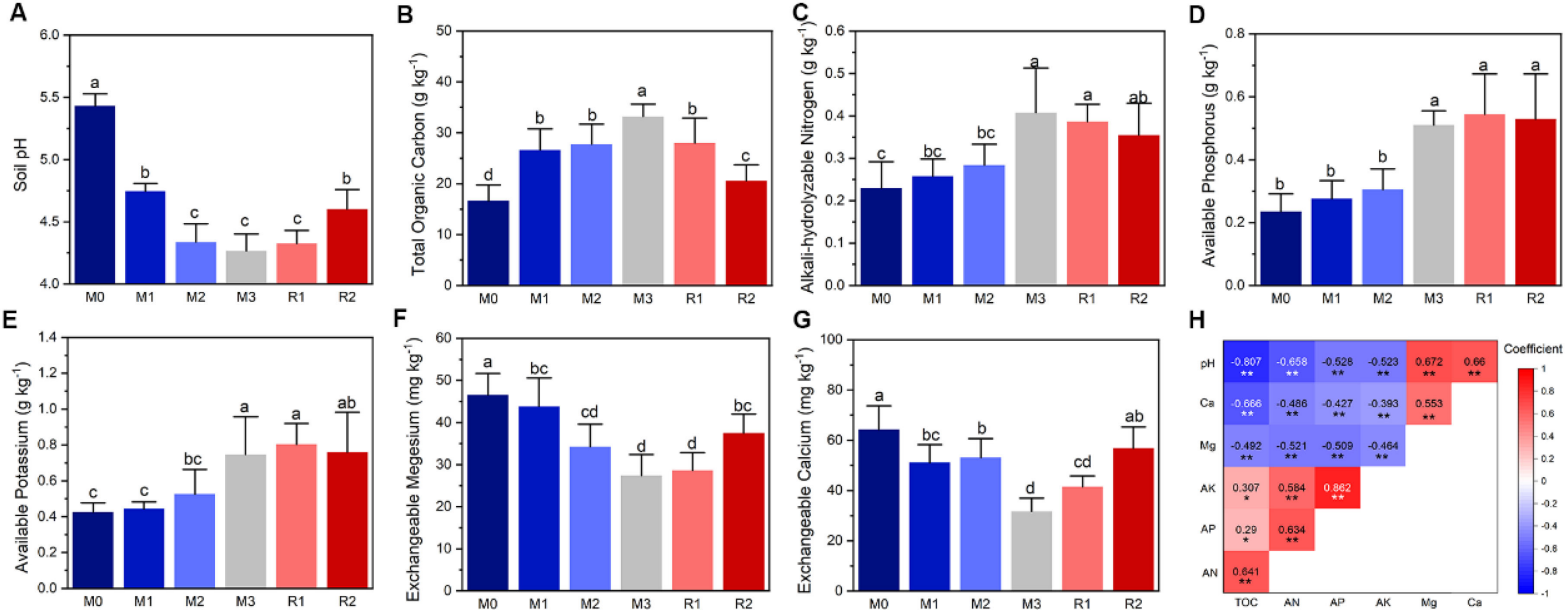
The dynamics of soil physiochemical properties and their relationship analysis. The lowercase letters in subfigures **(A–G)** indicate significant differences at the *P* < 0.05 level (Tukey’s HSD), the * and ** in subfigure **(H)** indicate significant correlation at the levels of *P* < 0.05 and *P* < 0.01, respectively. The Ca, Mg, AK, AP, and AN in subfigure **(H)** indicate exchangeable calcium, exchangeable magnesium, available potassium, available phosphorus and alkali-hydrolysable nitrogen, respectively. The M0 to M3, respectively, indicate mulched for 0–3 years and R1 and R2, respectively, indicate recovered for 1 and 2 years.

The results showed that soil pH, TOC, AN, TP, AK, AP, exchangeable magnesium, and calcium were all significantly changed with the increasing mulching ages (**Figure 1**). TOC, AN, AP, and AK showed an increasing trend that the value in M3 was nearly double of that in M0 (**Figures 1B–E**). But soil pH exchangeable magnesium and calcium lead a decreasing trend (**Figures 1A, F, G**). The exchangeable Mg decreased from M0 (46.56 ± 5.07 mg kg^−1^) to M3 (27.39 ± 5.00 mg kg^−1^) (**Figure 1F**) and exchangeable Ca similarly decreased from 64.25 ± 9.48 mg kg^−1^ (M0) to 31.65 ± 5.29 mg kg^−1^ (M3), (**Figure 1G**). Most of properties showed a partial recovery after the removal of mulching, except AP and AK (**Figures 1A–G**). Pearson correlation analysis found the factors were all significantly correlated (**Figure 1H**).

**Figure 2:**
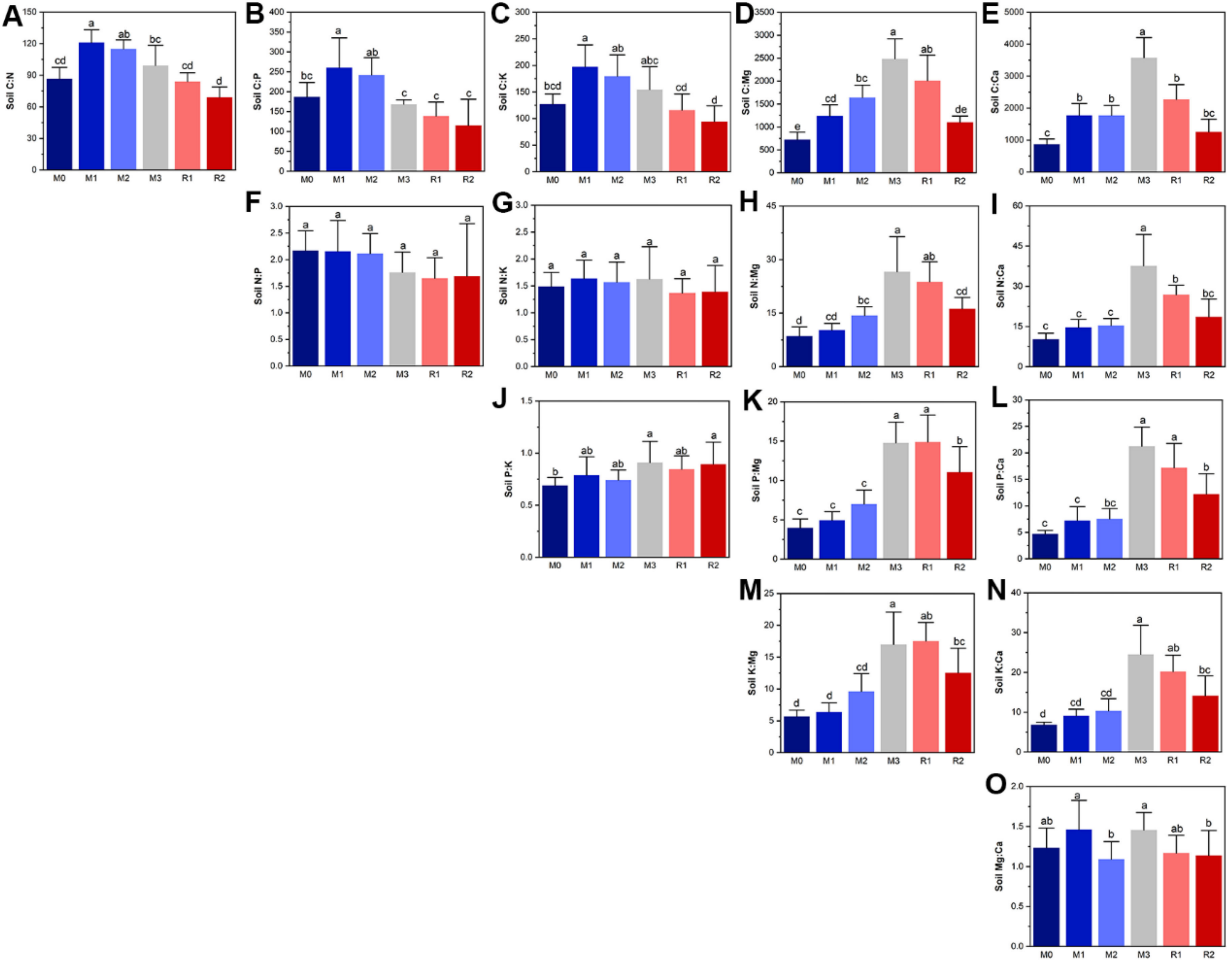
The subfigure **(A–O)** depicts the dynamics of soil nutrient stoichiometry. The lowercase letters indicate significant differences at the level of *P*<0.05 (Tukey’ HSD). The M0 to M3 respectively indicate mulched for 0 to 3 years and R1 and R2 respectively indicate recovered for 1 and 2 years.

The most prominent feature of soil nutrient stoichiometry dynamic was that the relative limitations of magnesium and calcium (C:Mg, N:Mg, P:Mg, K:Mg, C:Ca, N:Ca, P:Ca, and K:Ca) increased during mulching and partially recovered after mulch removal, which indicated the increased relative limitation of magnesium and calcium were induced by mulching (**Figures 2D, E, H, I, K–N**). As the ratios of P:Mg and P:Ca showed the greatest variance in magnitude, increasing from 3.97 ± 1.14 and 4.70 ± 0.66 (M0) to 14.76 ± 2.66 and 21.24 ± 3.64 (M3), respectively. The C:N, C:P, and C:K significantly increased in the first year of mulching, reaching 121.0 ± 12.13, 260.7 ± 74.77, and 197.3 ± 41.12, respectively. They then subsequently decreased until reaching the values of 69.10 ± 9.81, 114.8 ± 66.36, and 94.24 ± 29.65, respectively, in R2 (**Figures 2A–C**). P:K showed an increasing trend during the mulching period, with highest and lowest values of 0.909 ± 0.204 (M3) and 0.689 ± 0.077 (M0), respectively (**Figure 2J**). There were no significant variations in N:P and during the mulching period, the value of N:P was higher during M0–M3 than in the M3 to R2 stage (**Figure 2F**). No regular trends were observed for N:K (**Figure 2G**) and Mg: Ca (**Figure 2O**).

### 3.2 The influence of mulching on Lei-bamboo tissue nutrients and stoichiometry

**Figure 3:**
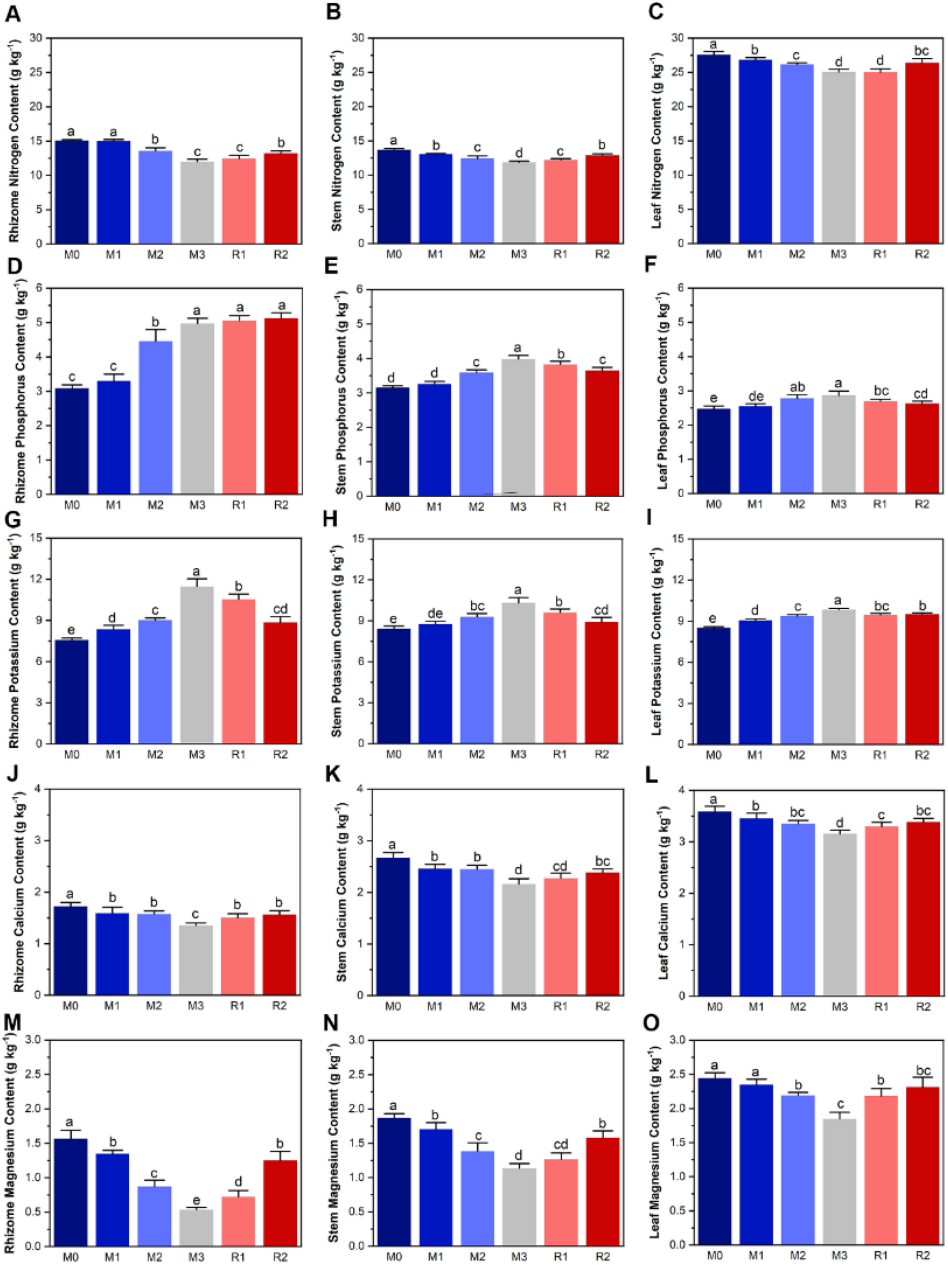
The subfigure **(A–O)** depicts dynamic of plant tissue nutrient. The lowercase letters indicate significant differences at the level of *P*<0.05 (Tukey’ HSD). The M0 to M3 respectively indicate mulched for 0 to 3 years and R1 and R2 respectively indicate recovered for 1 and 2 years.

The contents of nitrogen, phosphorus, potassium, calcium, and magnesium in Lei-bamboo tissue were significantly altered by mulching, and nearly all of them showed at least partially recovery (except for phosphorus in the rhizome) after mulch removal (**Figure 3**). The TP and TK in rhizome, leaf, and stem showed an increasing trend with mulching ages while the magnesium and calcium showed a decreasing trend (**Figures 3D–O**), which was similar to what happened in soil (**Figure 1**). However, the TN decreased during mulching years, which was opposite to its trend in soil (**Figures 1**, **3A–C**). The nitrogen content of rhizome, stem, and leaf significantly decreased from 15.06 ± 0.14 g kg^−1^ (M0) to 12.00 ± 0.37 g kg^−1^ (M3), from 13.67 ± 0.20 g kg^−1^(M0) to 11.84 ± 0.21 g kg^−1^ (M3) and from 27.57 ± 0.47 g kg^−1^ g kg^−1^ (M0) to 25.05 ± 0.43 g kg^−1^ (M3), respectively, and then recovered to 13.21 ± 0.35 g kg^−1^, 12.92 ± 0.19 g kg^−1^, and 26.43 ± 0.59 g kg^−1^ (R2), respectively (**Figures 3A–C**). Instead, Mg in rhizome, stem, and leaf were, respectively, 1.56 ± 0.12 g kg^−1^, 1.87 ± 0.06 g kg^−1^, and 2.44 ± 0.080 g kg^−1^ (M0), but the values in M3 decreased to only 34%, 60.7%, and 75.5% that of M0 before recovering to 1.251 ± 0.131 g kg^−1^, 1.582 ± 0.099 g kg^−1^, and 2.311 ± 0.146 g kg^−1^ (R2) (**Figures 3M–O**).

**Figure 4:**
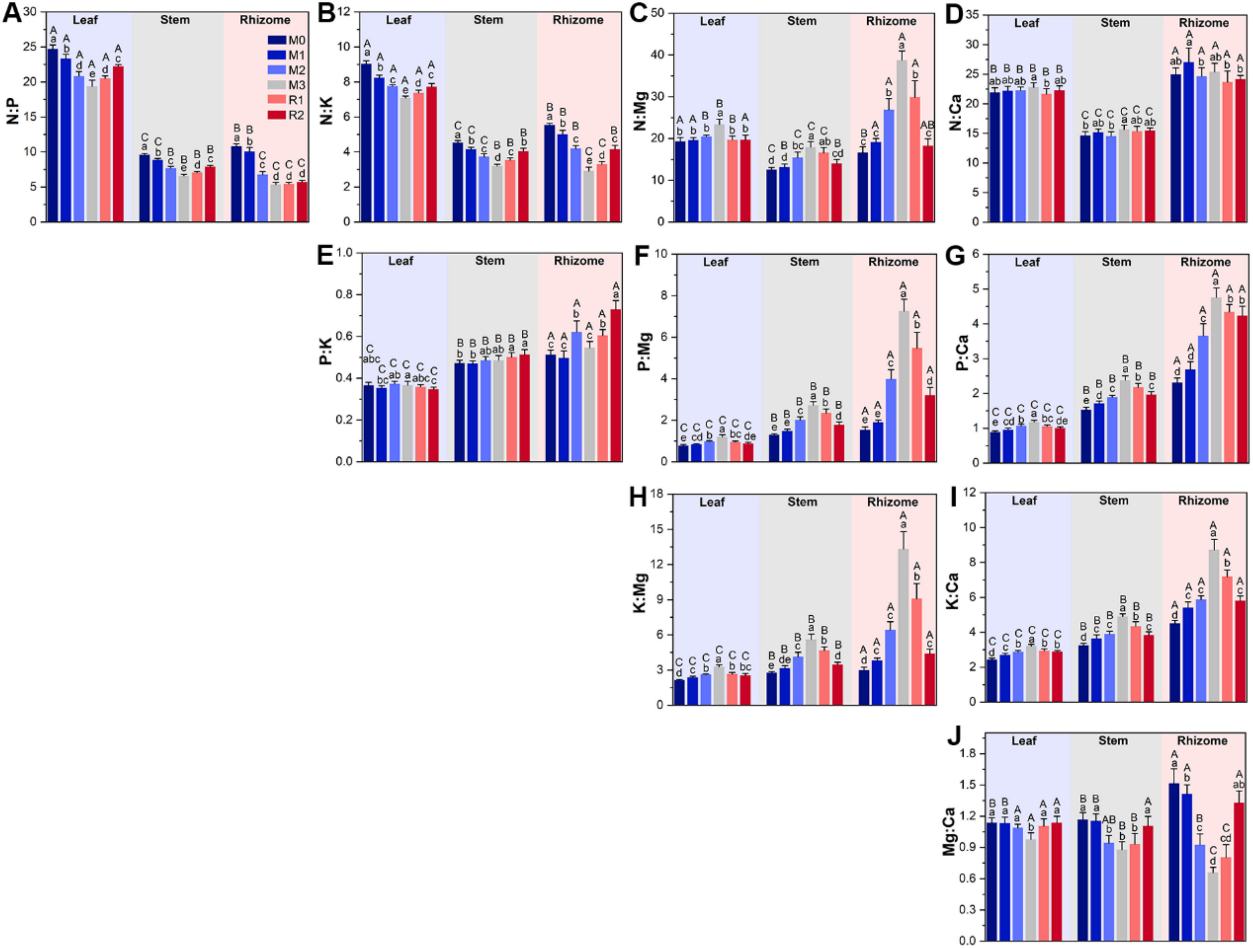
The subfigure **(A–J)** depicts dynamics of plant tissue nutrients stoichiometry. The lowercase letters indicate significant differences among the stages at the level of *P*<0.05 (Tukey’ HSD). The uppercase letters indicate significant differences among leaf, stem and rhizome at the level of *P*<0.05 (Tukey’ HSD). The M0 to M3 respectively indicate mulched for 0 to 3 years and R1 and R2 respectively indicate recovered for 1 and 2 years.

The results of plant tissue nutrients stoichiometry variation highlighted the magnified limitations of calcium and, especially, magnesium caused by mulching, and the greater magnitude of in variation in rhizome than stem and leaf (**Figure 4**). First, N:Ca, P:Ca, K:Ca, N:Mg, P:Mg and K:Mg of leaf, stem, and rhizome (except rhizome N:Ca) were increased by mulching, and decreased during the recovery period (**Figures 4C, D, F–I**). Rhizome P:Mg and K:Mg varied the most, increasing by nearly 4 and 3.5 times from M0 (1.54 ± 0.14 and 3.00 ± 0.25) to M3 (7.26 ± 0.57 and 13.34 ± 1.46), respectively (**Figures 4F, H**). Yet, mulching reduced N:P and N:K (**Figures 4A, B**). For instance, leaf N:P was 24.70 ± 0.60 in M0 and decreased to 19.39 ± 0.88 in M3, before increasing to 22.25 ± 0.24 in R2 (**Figures 4A, B**). The Mg: Ca was also decreased by mulching (**Figure 4J**). Second, the stoichiometry in rhizome was highest in rhizome, then stem and leaf (**Figure 4**). For example, the P:Mg of leaf and stem were only increased by 0.55 and 1.1 times, respectively, while K:Mg only increased by 0.54 and 1.0 times (**Figures 4F, H**), which was significantly lower than rhizome.

### 3.3 The influence of mulching on the richness and composition of the rhizosphere microbial community

**Figure 5:**
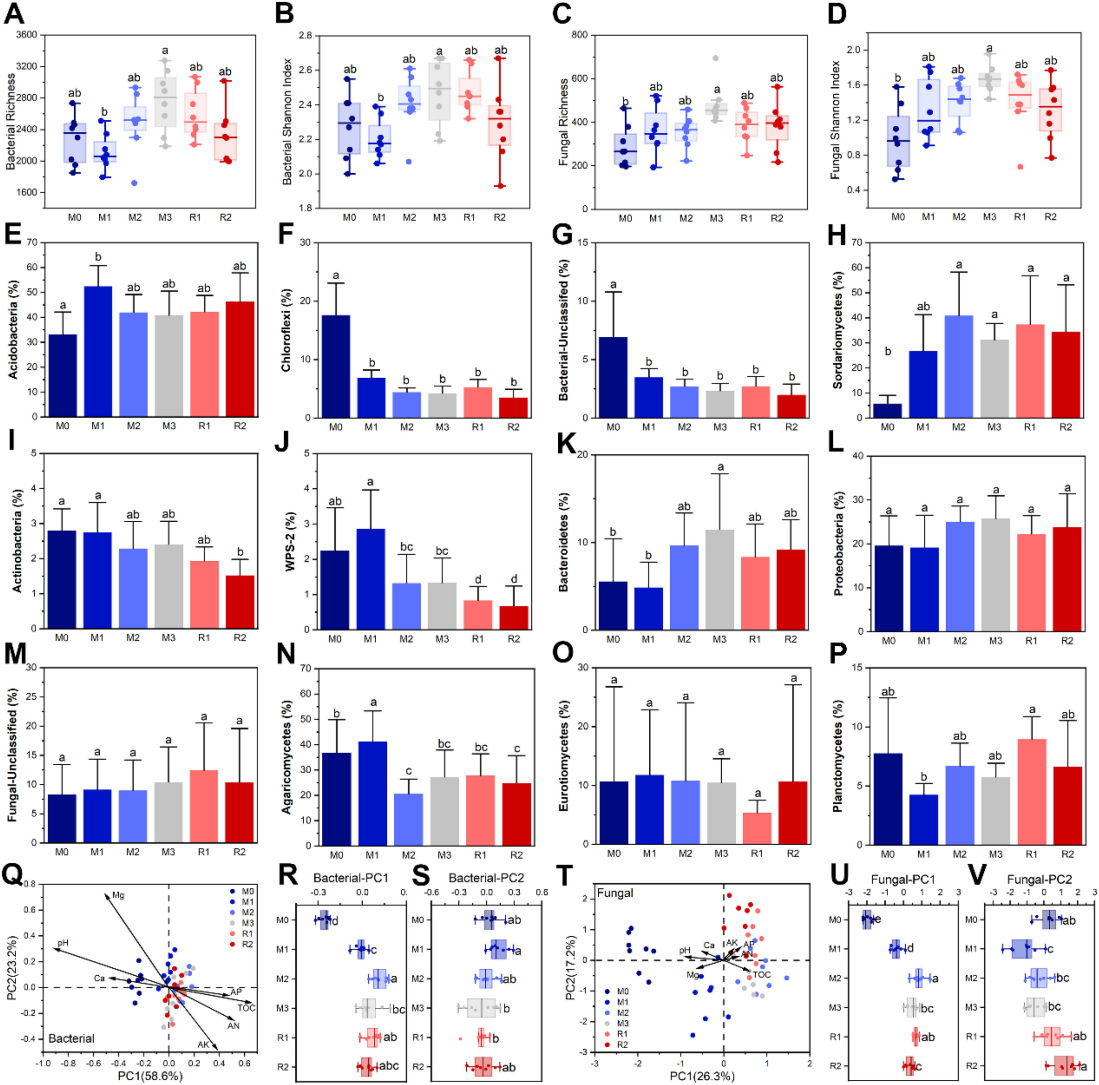
The α-diversity index and the β-diversity dynamics of the rhizosphere bacterial and fungal communities. Subfigures **(A–D)** depict the α-diversity index variation. Subfigures **(E–P)** depict the relative abundance variation of major bacterial phyla and fungal orders. Subfigures **(Q–V)** depict the RDA/CCA analyses of the bacterial and fungal community compositions at OTU level, respectively. The lowercase letters in subfigures indicate significant differences at the level of *P* < 0.05 (Tukey’s HSD). The M0 to M3 respectively indicate mulched for 0 to 3 years and R1 and R2, respectively, indicate recovered for 1 and 2 years. The TOC, Ca, Mg, AK, AP, and AN in subfigures **(Q, T)** indicate total organic carbon, exchangeable calcium, exchangeable magnesium, available potassium, available phosphorus and alkali-hydrolysable nitrogen, respectively.

Chao and Shannon indices of bacterial and fungal community in rhizosphere were both enhanced by mulching, but they were not significantly reduced after mulching removal. The Chao and Shannon indices of the bacterial community were lowest in M1 (2113 ± 223 and 2.20 ± 0.11, respectively). The Chao and Shannon indices increased to maximums of 2758 ± 390 and 2.47 ± 0.19 in M3 and then weakly fall back to 2325 ± 345 and 2.30 ± 0.22 in R2, respectively (**Figures 5A, B**). The Chao and Shannon indices of the fungal community were 288 ± 94 and 0.98 ± 0.37 in M0 before significantly increasing to 482 ± 91 and 1.67 ± 0.15 (M1) and declining back to 384 ± 108 and 1.32 ± 0.33 (R2), respectively (**Figures 5C, D**).

The results showed that mulching altered the RA of several taxa, but the effects were not consistent, and most of them were not recovered after mulching removal (**Figure 5**). The RA of several taxa was significantly changed after the first year but did not significantly change during the subsequent stages. These taxa included Acidobacteria (RA from M0 33.15 ± 9.00% to M1 52.48 ± 8.30%), Chloroflexi (RA from M0 17.60 ± 5.47% to M1 6.85 ± 1.36%), Unclassified Bacteria (RA from M0 6.94 ± 3.85% to M1 3.48 ± 0.75%) and Sordariomycetes (RA from M0 5.73 ± 3.33% to M1 26.72 ± 14.57%) (**Figures 5E–H**). The RA of Actinobacteria and WPS-2 decreased during the mulching and recovery periods, from 2.80 ± 0.62% and 2.24 ± 1.22% (M0) to 1.52 ± 0.46% and 0.67 ± 0.57% (R2), respectively (**Figures 5I, J**). The RA of Bacteroidete*s* increased from nearly 5% (M0 and M1) to nearly 10% (M2 and M3), then decreased slightly to 8.34 ± 3.77% and 9.21 ± 3.39% in R1 and R2, respectively (**Figure 5K**). The RA of Proteobacteria also increased from 19.60 ± 6.78% (M0) to 25.73 ± 5.23% (M3), before decreasing slightly to about 23% (R1 and R2) (**Figure 5L**). The RA of Unclassified Fungi gradually increased from 8.27 ± 5.13% (M0) to 12.41 ± 8.13% (R1), with a decrease in R2; however, no significant differences were observed among any of the stages (**Figure 5M**). Several taxa displayed no regular dynamic during mulching and recovery (**Figures 5N–P**). The maximum and minimum RA of Agaricomycetes were 41.21 ± 12.13% (M1) and 20.58 ± 5.81% (M2), respectively (**Figure 5N**). The RA of Eurotiomycetes was nearly same for every stage (about 10%) except R1 (5.35 ± 2.13%) (**Figure 5O**). The RA of Planctomycetes was lowest in M1 (4.25 ± 0.96%) and highest in R1 (8.95 ± 1.92%) (**Figure 5P**).

RDA analysis showed that bacterial OTU-level composition was significantly altered by mulching, especially from M0 to M1 (**Figure 5Q**), as the PC1 varied significantly from −0.257 ± 0.035 (M0) to −0.011 ± 0.039 (M1) (**Figure 5R**), but no significant differences were found among M3, R1, and R2, for both PC1 and PC2 (**Figures 5R, S**). The ANOSIM analysis also found that the *P*-values for between M3 and R1 and for between R1 and R2 were 0.05 and 0.144, respectively (**Supplementary Table S1**). These indicated that the composition of the rhizosphere bacterial community had not recovered after mulch removal. The magnitudes of contribution made by the environmental factors to bacterial composition was pH > TOC > Mg > AK > AN > Ca > AP. (**Figure 5Q**) Fungal community composition at the OTU level was also significantly shaped by mulching (**Figure 5T**). The compositional variation of fungi was greater than that for bacteria in the first year, as not only did PC1 significantly vary, from −1.991 ± 0.236 (M0) to −0.374 ± 0.275 (M1), but PC2 also varied significantly, from 0.288 ± 0.599 (M0) to −1.193 ± 0.826 (M1) (**Figures 5U, V**). After mulch removal, PC1 did not recover, but PC2 showed a partial recovery, to 1.224 ± 0.728 (R2), which was significantly higher than in M3. ANOSIM analysis found that the *P*-value for between M3 and R1 and for between R1 and R2 were, respectively, 0.025 and 0.016, both higher than 0.01 (**Supplementary Table S1**). This indicated that the fungal community was only weakly recovered. The magnitude of contribution by environmental factors to fungal composition was pH > Mg > TOC > AP > Ca > AK > AN (**Figure 5T**).

### 3.4 The influence of mulching on the rhizosphere microbial community network

**Figure 6:**
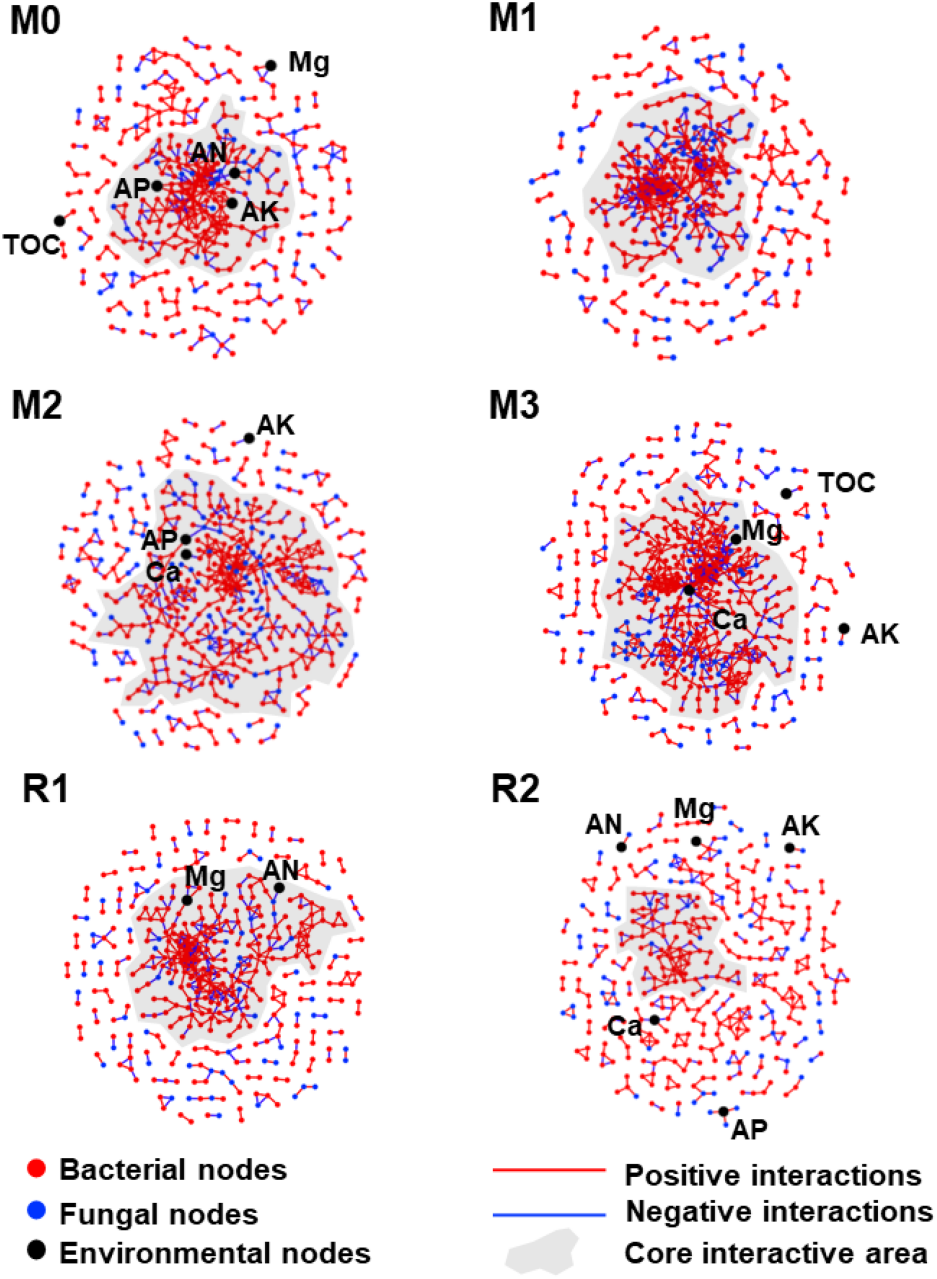
The co-occurrence networks during the mulching and recovery periods. The red, blue, and black dots indicate bacterial, fungal, and environmental members, respectively. The red and blue lines indicate positive and negative interactions, respectively. The shaded areas indicate the collection of core interactive modules. The subfigures M0 to M3, respectively, indicate mulched for 0 to 3 years, and R1 and R2, respectively, indicate recovered for 1 and 2 years. The TOC, Ca, Mg, AK, AP, and AN indicate total organic carbon, exchangeable calcium, exchangeable magnesium, available potassium, available phosphorus and alkali-hydrolysable nitrogen, respectively.

The network became more clustered and interactive with increasing mulching age and looser during recovery. Moreover, AP, AK, and AN were found in the core interactive area of the M0 network, while AP and Ca were found in the core interactive area of M2, and magnesium and calcium were found in the core interactive area of M3. The results indicated that mulching enhanced the complexity of the rhizosphere microbial network and led microbes to focus more on magnesium and calcium (**Figure 6**).

**Figure 7:**
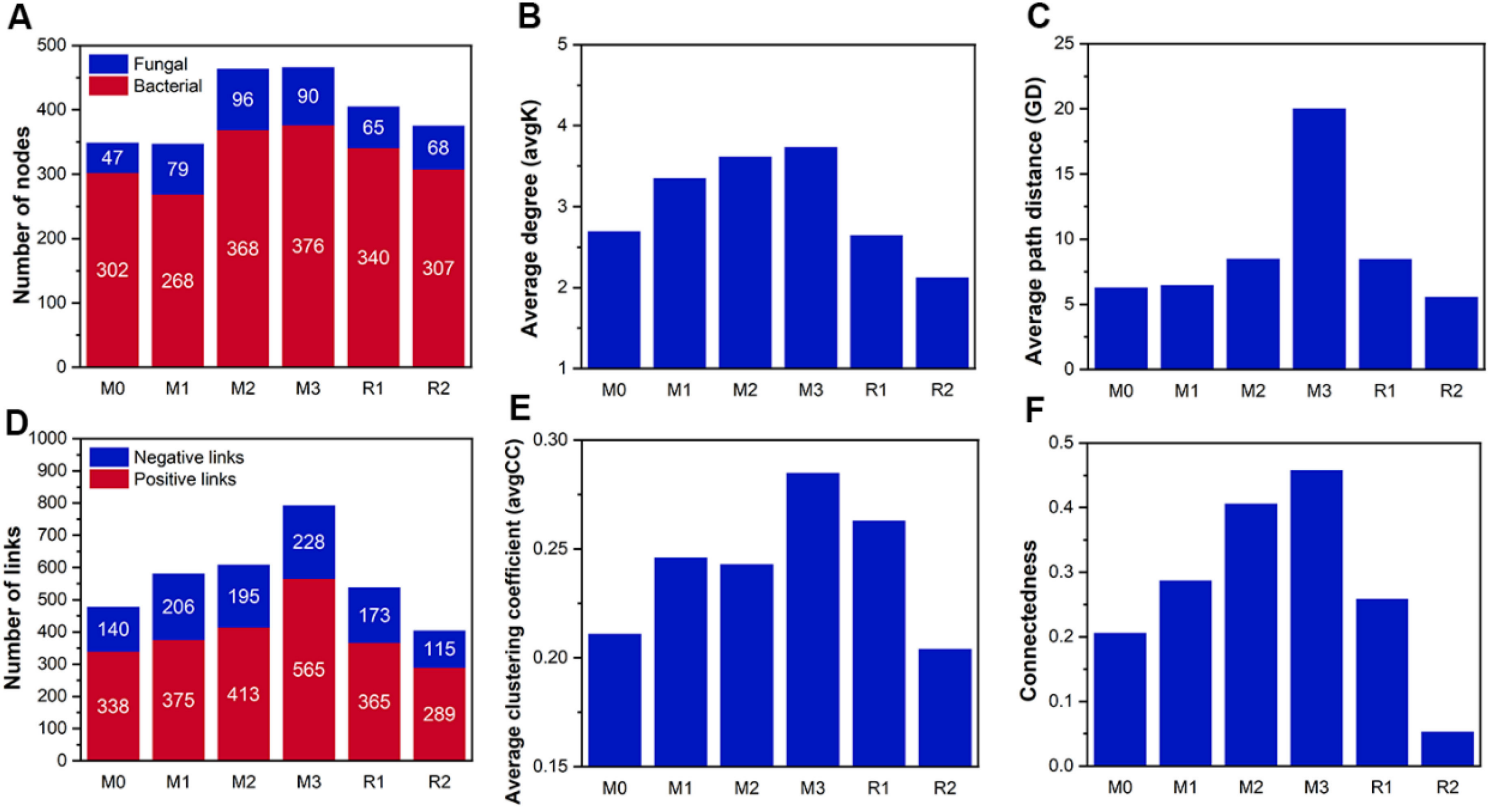
The parameters of co-occurrence networks during the mulching and recovery periods. The M0 to M3, respectively, indicate mulched for 0 to 3 years, and R1 and R2, respectively, indicate recovered for 1 and 2 years. The blue and red part in subfigure **(A)** indicate fungal and bacterial node numbers, respectively. The blue and red parts in subfigure **(D)** indicate negative and positive links, respectively. The subfigure **(B, C, E, F)** depicts the average degree, the average path distance, the average clustering coefficient and the connectedness of networks, respectively.

The number of network nodes gradually increased from 349 (bacterial 302 and fungal 47 in M0) to 466 (bacterial 376 and fungal 90 in M3), then decreased to 375 (bacterial 307 and fungal 68 in R2). The fungal nodes had a higher increment proportion than bacterial nodes (**Figure 7A**). The link number was also enhanced by mulching, increasing from 478 (140 negative and 338 positive in M0) to 793 (228 negative and 565 positive in M3), before decreasing to 404 (115 negative and 289 positive in R2) (**Figure 7D**). The avgK, GD, avgCC, and connectedness also increased with mulching age, respectively, reaching maximums of 3.734, 20.31, 0.285, 0.458 in M3, which was about 1.38, 1.35, 3.17, and 2.22 times that of M0 (**Figures 7B, C, E, F**). The recovery period decreased network parameters to a level that were sometimes even lower than that of stage M0. The avgK and connectedness in R2 were, respectively, 2.126 and 0.053, which was lower than in M0 (2.693 and 0.206). This indicated that the size and the complexity of the network were enhanced by mulching. Also, the number of key nodes in stages M0 to R2 were 3, 2, 3, 6, 2, and 1, and the proportion of Proteobacteria in key nodes increased with mulching, indicating a potential shift in network functional preference (**Supplementary Figure S1**).

### 3.5 The mechanism by which mulching induces Lei-bamboo calcium and magnesium deficiency

**Figure 8:**
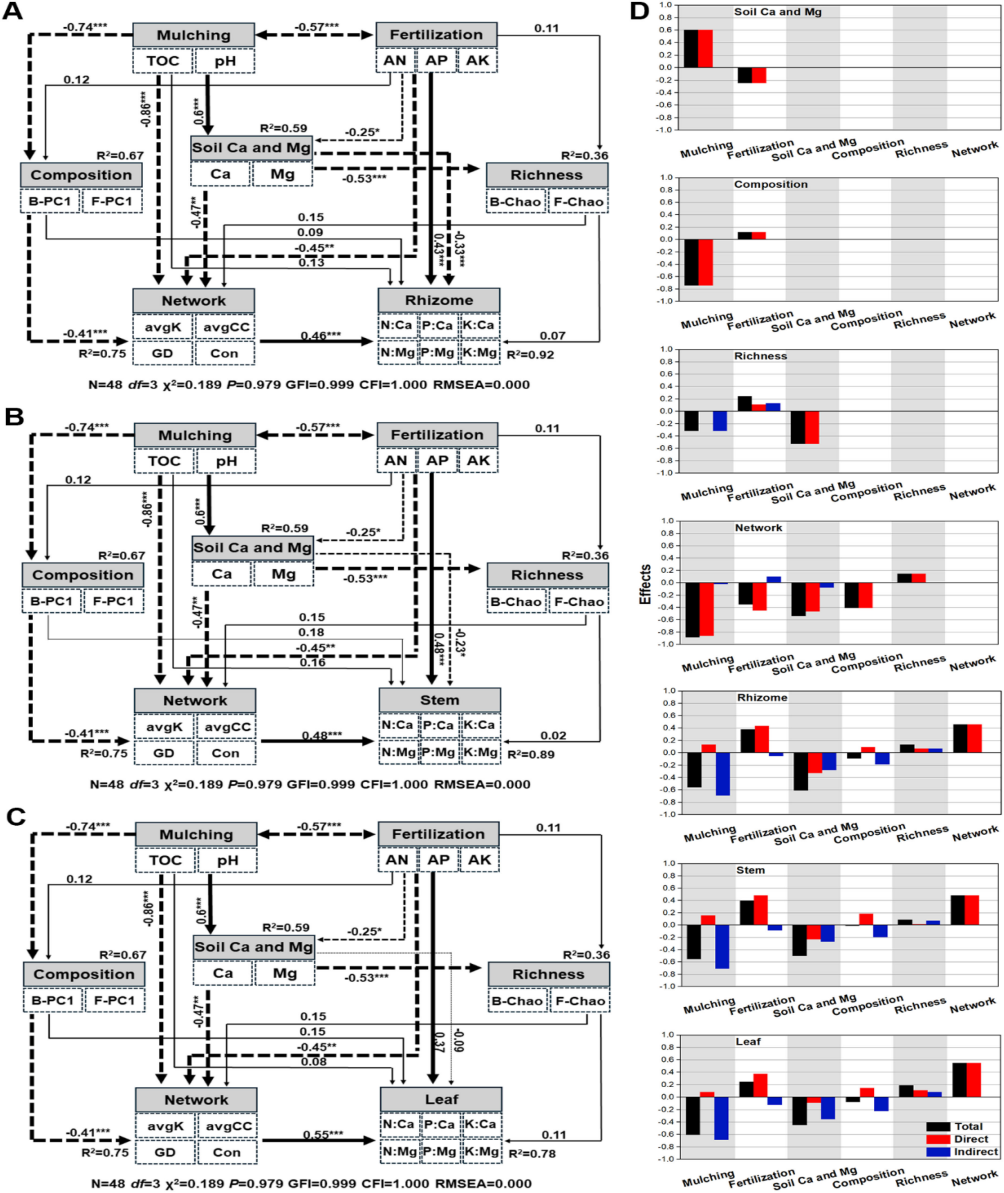
The structural equation model resolving the mechanisms of mulching on Lei-bamboo tissue calcium and magnesium limitation. **(A–C)** Explain rhizome, stem, and leaf, respectively. Subfigure **(D)** shows the effects offered by the factors. The solid and dashed lines indicate negative and positive correlations, respectively. The *, **, and *** indicate significant correlations at the level of *P* < 0.05, *P* < 0.01, and *P* < 0.001, respectively. The TOC, Ca, Mg, AK, AP, and AN indicate total organic carbon, exchangeable calcium, exchangeable magnesium, available potassium, available phosphorus, and alkali-hydrolysable nitrogen, respectively. The avgK, GD, avgCC, and con indicate the average degree, the geographic distance, the average clustering coefficient, and connectedness of the structure equation modeling, respectively. B-PC1 and F-PC1 indicate the first axis coordinate of bacterial and fungal community composition of RDA/PCA analysis, respectively. B-Chao and F-Chao indicate bacterial and fungal Chao index, respectively.

Both mulching and fertilization influenced soil exchangeable magnesium and calcium content, mulching offered 0.603 standard total effects (STEs), which was nearly 2.4 times the magnitude of fertilization (−0.247) (**Figure 8D**). Mulching offered −0.742 STEs to microbial community composition, which was about 5.2 times higher than fertilization (0.119). The exchangeable calcium and magnesium offered the greatest STEs to microbial richness (−0.527), while mulching and fertilization offered only −0.318 and 0.24, respectively. Mulching offered −0.887 STEs to network complexity, which was higher than calcium and magnesium (−0.543) and microbial composition (−0.409) offered. These results showed that mulching had a greater impact on the soil exchangeable calcium and magnesium and microbial community properties than fertilization (**Figure 8D**).

The mulching offered, respectively, −0.56, −0.556, and −0.606 STEs to the calcium and magnesium limitation of rhizome, stem, and leaf, and soil calcium and magnesium respectively offered −0.612, −0.505, and −0.45 STEs, while fertilization only offered 0.38, 0.397, and 0.247 STEs, respectively. To the biotic factors, network complexity offered 0.459, 0.484, and 0.549 STEs, respectively, to the calcium and magnesium limitation on rhizome, stem, and leaf, which was much higher than composition and richness offered (**Figure 8D**). The results indicated that mulching, rather than fertilization, was more influential on the calcium and magnesium limitation in Lei-bamboo tissue, mainly by changing soil Ca and Mg content and by regulating network interactions of the rhizosphere microbial community (not composition or richness).

## 4 Discussion

### 4.1 The soil physiochemical properties variation under mulching

Soil TOC and acidification were enhanced by mulching (**Figures 1A, B**), and TOC and pH were highly correlated (**Figure 1A**). During Lei-bamboo mulching, the soil is covered by a layer of wet organic material more than 25 cm thick, that includes straw and rice husks, to enhance the soil temperature via the metabolic heat released by microbes within the mulch (Wei et al., 1994; Wang et al., 2017). Due to the considerable thickness and water content of the materials, anaerobic fermentation will occur in the anoxic ozone (Qian et al., 2022; Mbukwa et al., 2023), leading to the production of organic acids (Lim et al., 2008; Dionisi and Silva, 2016; Li et al., 2023). The downward transportation of these organic acids via rainfall or irrigation will lower soil pH. Excessive fertilizer, such as urea, will also lead lower soil pH, because of the free protons released when ammonia is oxidized to nitrate (Wang et al., 2023c). Thus, the over application of fertilizer may contribute to the soil acidification of intensively cultivated Lei-bamboo forests (**Figures 1C–E**). Meanwhile, in the current study, soil pH was partially recovered after removal of the mulch layer, which indicated that the organic acid released by mulching, rather than fertilization, was the main cause of soil acidification.

The contents of soil exchangeable Ca and Mg were significantly lowered by mulching (**Figures 1F, G**), which was closely related to the enhanced TOC and acidification. Ca and Mg are metals belonging to Group II of the periodic table, and exhibit similar chemical properties and geological processes in soil (Krám et al., 2019). Ca and Mg are mostly enriched in the early and middle stages of magma crystallization, and stored in soil parent materials in the form of metasilicate-type Ca-Mg salts, Ca-Mg olivines, and so forth (Su et al., 2019). Ca and Mg will be released into soil solution with the weathering of soil parent materials, and part of the Ca and Mg in solution will combine with the negatively charged surfaces of soil particles and organic matter, which then become the forms available to plants (Whittinghill and Hobbie, 2012). Climatic factors are determinative to the geochemical cycles of soil Ca and Mg (Boy and Wilcke, 2008; Whittinghill and Hobbie, 2012). In the dry areas, Ca and Mg are prone to form precipitates that are recalcitrant to leaching and weathering (Boy and Wilcke, 2008). While in wet and warm areas, like the locations where Lei-bamboo grow, soil Ca and Mg are vulnerable to leaching (Whittinghill and Hobbie, 2012), while the silica-aluminate parent materials there are recalcitrant to weathering for the release of new Ca and Mg, which led to a lower content of soil exchangeable Ca and Mg (Prietzel et al., 2021). The deficiency of Ca and Mg can be amplified by mulching. The organic matters produced by mulching materials absorb the dissolved Ca and Mg cations, as well as accelerate the leaching of Ca and Mg when they are transported downward by rainfall and irrigation (Adhikari et al., 2019). The increased levels of protons in soil solution compete with Ca and Mg ions for the same negatively charged positions on soil particles, which then force the originally attached Ca and Mg to release as free cations and become more vulnerable to leaching (Clarholm et al., 2015). Thus, through enhanced TOC and acidification, mulching accelerates the leaching of Ca and Mg in soil during intensive cultivation of Lei-bamboo forests, and further lowers soil exchangeable Ca and Mg content.

### 4.2 The RMC composition dynamic under mulching

RMC composition was mainly altered by TOC and pH (**Figures 5Q, T**). Fermentation in the mulching layer releases intermediate metabolites, most of which are small molecular, labile organic carbon compounds that can be easily utilized by microbes. The enhanced labile organic carbon enriched r-strategy taxa, such as Proteobacteria (Wang et al., 2021) (**Figure 5L**), but lowered the RA of K-strategy taxa, such as Actinobacteria (Ho et al., 2017) (**Figure 5I**) and WPS-2 (Sheremet et al., 2020) (**Figure 5J**), which are capable of degrading recalcitrant organic carbon compounds. Lowered soil pH favors acidophilic groups, including the Acidobacteria (Rousk et al., 2010) (**Figure 5E**) and Sordariomycetes (Xing et al., 2022) (**Figure 5H**). It is unclear why community composition did not recover even when the soil physicochemical properties were partially ameliorated. This is possibly connected to the long-term degradation mechanisms of intensively cultivated Lei-bamboo. In contrast to short-term yield reduction, long-term reduction cannot be reversed by removing the mulching alone but requires complete removal and replacement of the soils and replanting with new bamboo, which is costly and, as such, has become a core problem of Lei-bamboo intensive cultivation (Wei et al., 1994). There are several possible reasons. First, the heavy metals that are potentially co-applied with the fertilizers may then accumulate in the soils and plants (Wei et al., 1994). Second, the release of labile organic carbon could induce a “priming effect,” reshaping the soil’s organic carbon composition as well as microbial community composition (Zhang et al., 2019; Shi et al., 2023). Third, long-term intensive cultivation likely caused the deficiency of certain functionally important micro-elements (Kumar et al., 2020). Last, while the α-diversity recovered, the β-diversity did not (**Figure 5**), which indicated that the contribution of rare species to the RMC composition had been weakened. These rare species may play key roles in certain ecological functions (Jousset et al., 2017; Wang et al., 2023a). Thus, rare species related compositional variation and functional disability might directly drive the long-term reduction in bamboo yield. The mechanisms mentioned above are potential factors that lead to long-term and irreversible changes in the soil microbial community as well as the degradation of Lei-bamboo cultivation but require further confirmation.

It is worth noting that Mg also contributed considerably to RMC composition, even more than AN, AP, and AK did (**Figures 5Q, T**), which indicated the undervalued importance of Mg in Lei-bamboo ecosystems. Mg could affect the RMC composition in both direct and indirect ways. On the one hand, Mg deficiency might directly enhance the RA of microbial taxa that possess high tolerance to this type of stress. For example, some microbes have a higher capability to solubilize Mg bearing minerals (Silverman and Munoz, 1980; Pokrovsky et al., 2021), others, can absorb the free Mg ions by zooglea, or can store Mg within cell compartments, such as the cell wall (Wang et al., 2019). On the other hand, Mg deficiency might offer indirect effects by altering the availability of other factors. For example, the application of Mg to acidic soil reduces the soil adsorption of phosphorus, thus lowering the relative limitation of phosphorus and altering microbial composition at the enzymic level (Lei et al., 2024). The addition of Mg to rice fields immobilizes Mn, As, and Cd in soil, lowering their potential toxicity to the microbial community (Zhang et al., 2023; Sun et al., 2024).

### 4.3 The variation on RMC network under mulching

As mulching age increased, magnesium and calcium also shaped microbial interactions as they gradually appeared in the core interactive areas of the ecological network (**Figure 6**). The network was constructed from functional modules, which are organized by microbial taxa that possess higher functional connections, including nutrient mineralization and acquisition (Deng et al., 2012). The RMC can alter member interactions according to the variation of specific nutrient limitations, using the core module to remediate the most limiting nutrient, and thus meet the demands of the whole community or host plants (Deng et al., 2012; Zhou et al., 2022). The RMC often organize the network center around phosphorous when the host shrub has high phosphorus demands, but will organize around nitrogen when the host grass has high-nitrogen demands (Zhou et al., 2022). Hence, the data in this study support that the magnesium and calcium limitation of the rhizosphere led a higher microbe functional emphasis in relation to those elements.

Mulching also increased the complexity and interactivity of the RMC network (**Figures 6**, **7**), which indicated a weakened functional dependency between RMC and roots. When facing stress, microbes tend to increase the expression of resistance related genes but decrease the expression of communication and interaction related genes, which reduce the number of interactions, leading to network simplification (Wang et al., 2018b; Zhou et al., 2021). The enhanced interactivity observed in the current study indicated that mulching introduced a more hospitable habitat rather than stresses to the RMC (Hernandez et al., 2021), even though it led to undesirable reduction in bamboo yield (Wei et al., 1994; Wang et al., 2017). Before mulching, the RMC received organic compounds from host plants and reciprocated with inorganic nutrients (Ling et al., 2022). However, the considerable labile organic carbon input from mulching reduced the RMC’s dependency on organic carbon exudates from the roots (Huang et al., 2014), which weakened the mutualistic interactions between microbes and host (Koide and Li, 1990; Hou et al., 2021). The RMC can release organic acids and chelating agents to improve the mobility of metal nutrients (Pokrovsky et al., 2021), and several taxa can use hyphae to break soil aggregates and expose the mineralized nutrients inside (Hou et al., 2021). Thus, the weakened mutualism in turn possibly hampered microbial assistance to host magnesium and calcium uptake.

Even though network parameters recovered after removal of the mulch (**Figures 6**, **7**), the community composition did not (**Figures 5R, S, U, V**), which indicated that network complexity and interactivity, rather than community composition, may be a better indicator for host dominance. For example, *Tricholoma matsutake* was marginalized from its RMC network due to climate change, which reduced the yield of *T. matsutake* and lead to the risk of regional extinction (Zhou et al., 2021). In high richness systems with high functional redundancy (Louca et al., 2018), community members can change their interactive pattern to meet host demands (Averill et al., 2022), and a well-organized RMC network is highly associated with a better rhizosphere multifunctionality, including magnesium and calcium solubilization and assimilation (Chen et al., 2022; Wang et al., 2023b; Zhai et al., 2024). Thus, improving the network structure of the RMC is a potential method to alleviate magnesium and calcium limitations.

### 4.4 The magnesium and calcium limitations on Lei-bamboo induced by mulching and suggestions for cultivation

In this study, it was found that the magnesium and calcium limitations of Lei-bamboo were enhanced by mulching (**Figures 4C, D, F–I**). Mulching probably interferes with calcium and magnesium acquisition of Lei-bamboo in two ways. First, by reducing the content of exchangeable magnesium and calcium, which directly decreased the potential quantity available for assimilation (Tang and Luan, 2017) (**Figure 8**). Second, by altering RMC network interactivity and weakening the functional dependency between RMC and roots, which further enhanced the difficulties of Lei-bamboo to take up magnesium and calcium (Pokrovsky et al., 2021) (**Figure 8**).

The increasing magnitude of magnesium and calcium limitation in Lei-bamboo is rhizome > stem > leaf (**Figure 4**), which indicated that magnesium and calcium deficiency led to a physiological response by the plant. Plants will preferentially supply the leaves with limited nutrients to ensure the stability of photosynthesis, which can improve individual competence under nutrient deficiency (Farhat et al., 2016). Moreover, the nitrogen content in Lei-bamboo was reduced despite increased AN in the soil (**Figure 3A**), which supported that nitrogen assimilation might be hampered by magnesium and calcium limitation. Mg can reduce nitrogen loss through decreased N_2_O emissions and nitrate leaching, enhancing the available nitrogen pool for crops (He et al., 2023). Mg also mitigates alumina toxicity in acidic soils and improves crop nitrogen uptake by roots (Lyu et al., 2023). Mg accelerates the leaf nitrogen storage in the form of amino acids (He et al., 2023). The application of Mg fertilizer can even double the effects of nitrogen fertilizers in intensively cultivated peanut fields (Gao et al., 2024b). These studies show that Mg can affect crop nitrogen utilization through the regulation of the soil nitrogen cycle, root assimilation, and magnesium transportation and storage. Ca was reported to possess similar functions (Lian et al., 2022; Yang et al., 2023; Gao et al., 2024a). Thus, the limitation of magnesium and calcium can induce yield degradation by hampering the utilization of other nutrients.

Considering that while AN, AP, and AK increased with mulching age (**Figures 1C–E**), but yield decreased, we suggest the application of nitrogen, phosphorus, and potassium had far exceeded the true demands of Lei-bamboo. More attention should be paid to the dynamics and limitations of magnesium and calcium, as well as other medium-quantity nutrients and micronutrients (Kumar et al., 2020; Ahmed et al., 2024). Over fertilization reduces utilization efficiency, increases production cost, and leads to the pollution and eutrophication of underground water, rivers, and lakes (Wang et al., 2018a; Tian et al., 2022; Huang et al., 2023). Excessive nutrients give the wrong signals to roots, misleading them to believe that nutrient demands could be meet by fewer roots (Chen et al., 2023), resulting in ill-developed roots and RMCs that in turn impedes the uptake of magnesium and calcium (Gui et al., 2013, 2018).

In this study, the enhanced TOC and acidification induced by mulching were the fundamental reasons for Lei-bamboo magnesium and calcium limitation, with the deficiency of magnesium and calcium in soil as potential direct reasons, and the altered RMC network considerably magnifying the effects of soil magnesium and calcium deficiency (**Figure 8**). Hence, we suggest to find alternative mulching materials that will not release organic acids (Wei et al., 1994; Li et al., 2023), or the application of biochar to buffer the acidification and increase the number of potential binding sites for free magnesium and calcium ions (Zhao et al., 2020; Zhang et al., 2023). We strongly recommend the application of CaO (Li et al., 2019) and MgO (Zhang et al., 2022) immediately after mulching as this inexpensive method can sterilize the pathogens enriched during mulching, increase soil pH, and directly supply magnesium and calcium to soils. Last, an effective microbial fertilizer to prevent the weakening of functional mutualism between RMC and roots is also an ecofriendly method (Huang et al., 2014).

## 5 Conclusion

As the mulching age increased, the TOC, AN, AP, and AK were enhanced but the exchangeable magnesium and calcium were reduced, maybe due to the acidification caused by organic acid released from mulching layer. This was a direct reason for the increased Ca and Mg-related stoichiometry (N:Mg, etc) in soil and Lei-bamboo tissue. Meanwhile, mulching increased network interactivity of RMC, a sign of weakened mutualism between RMC and root, which possibly in turn hampered the uptake of magnesium and calcium in root and amplified the magnesium and calcium limitation on Lei-bamboo tissue. This research highlighted the potential limitation on magnesium and calcium rather than usually fertilized nitrogen, phosphorus and potassium, which offered practical information for balancing fertilization on Lei-bamboo mulching cultivation and might help preventing short-term yielding degradations and boosting sustainable development of Lei-bamboo forest.

## Data Availability

The raw sequencing data are available in NCBI Sequence Read Archive of PRJNA1114674 (bacterial) and PRJNA1114879 (fungal).
