# Supplementary material for: Network and stoichiometry analysis revealed a fast magnesium and calcium deficiency of mulched Phyllostachys violascens
Source: Front Plant Sci. 2024 Nov 27;15:1492137. doi: 10.3389/fpls.2024.1492137 (PMC11632225; doi:10.3389/fpls.2024.1492137)

Table S1. The *P*-value of ANOSIM-test for bacterial and fungal communities at the OTU level

|  | Bacterial | M0 | M1 | M2 | M3 | R1 |
| --- | --- | --- | --- | --- | --- | --- |
|  | M1 | 0.001 |  |  |  |  |
|  | M2 | 0.003 | 0.003 |  |  |  |
| Bacterial | M3 | 0.001 | 0.003 | 0.002 |  |  |
|  | R1 | 0.001 | 0.001 | 0.001 | 0.005 |  |
|  | R2 | 0.001 | 0.001 | 0.044 | 0.008 | 0.144 |
|  | M1 | 0.002 |  |  |  |  |
|  | M2 | 0.001 | 0.001 |  |  |  |
| Fungal | M3 | 0.002 | 0.001 | 0.026 |  |  |
|  | R1 | 0.001 | 0.001 | 0.001 | 0.025 |  |
|  | R2 | 0.001 | 0.001 | 0.002 | 0.14 | 0.016 |

Figure S1. The functional key nodes of different stages


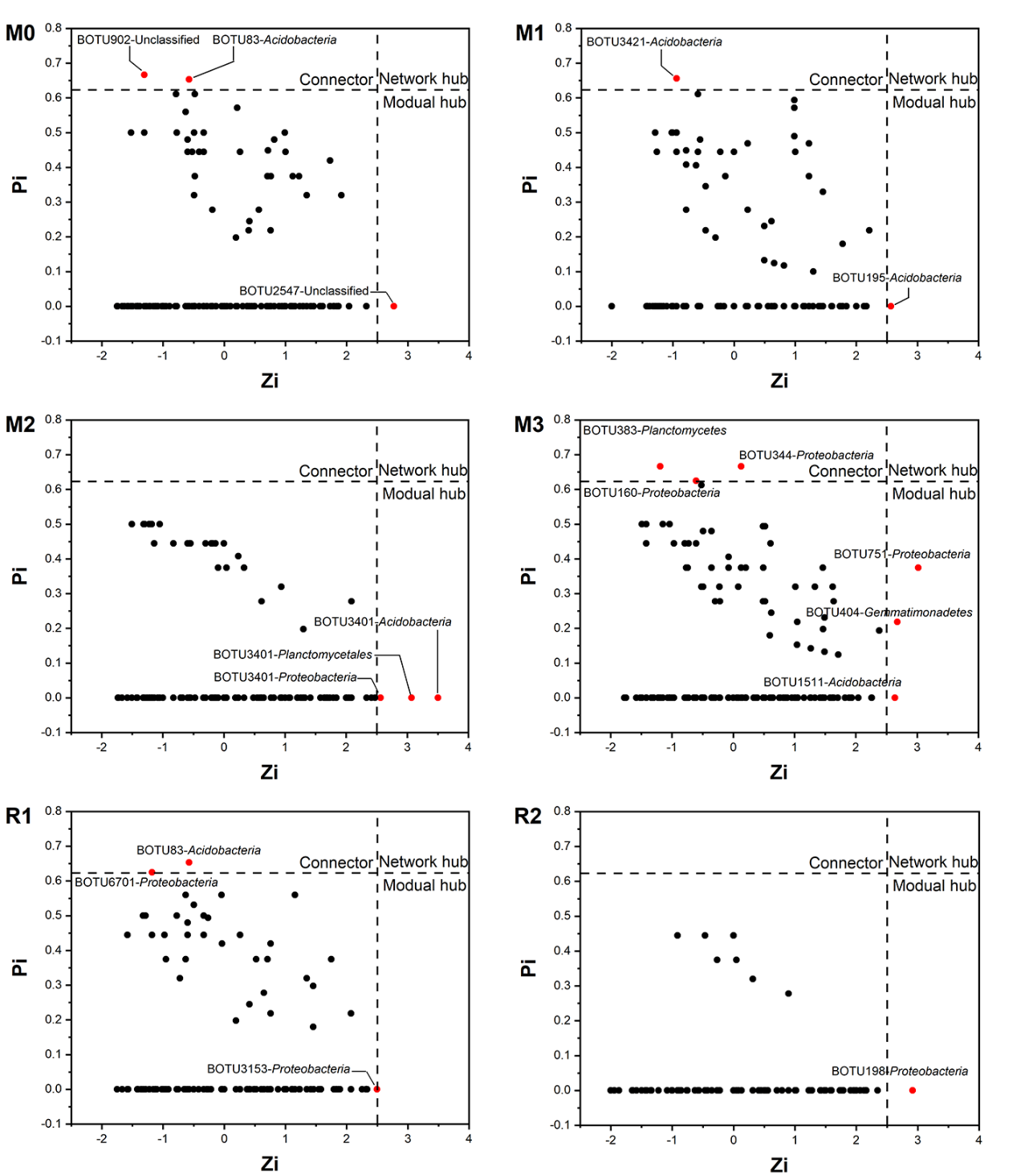

Supplement: Supplementary file 1 [file DataSheet1.docx]
